# Ununited Fracture Cured by Pressure

**Published:** 1828-08

**Authors:** T. H. Wright


### MISCELLANEOUS INTELLIGENCE.

---

### ANALYTICAL AND ORIGINAL.

1. *Ununited fracture cured by Compression.*—Dr. Thomas H. Wright, Physician to the Baltimore Alms-house Infirmary, in the 4th No. of the *American Journal of the Medical Sciences*, has published several cases, which go to exemplify and sustain the influence of Compression in cases of ununited fracture.

Case 1st. was that of a carpenter who fell from his scaffold and suffered an oblique fracture of the lower part of the tibia, a fracture of the fibula, and a projection of the end of the former bone through a ragged wound of the integuments two inches in extent. Three or four months after the accident, the bones were ununited and all signs of ossific orgasm were gone, Dr. W. then determined on trying pressure.

"The simple, efficient, and surgical mode of making pressure by the tourniquet, since successfully practised by Mr. Brodie, did not occur to me. A strong, soft, double napkin was passed under the leg, extending from below the ancle, to the head of the tibia, pad-compresses of suitable size, were laid, which, when braced by the general bandage, acted as counter-wedges on the line of fracture. The bandage or napkin first laid, was drawn firmly around the limb, and confined at short spaces; at first by large pins, and afterwards by tacks. It was thus made to give uniform support and a regulated pressure through the compresses on the line of fracture, and to adapt itself closely to the contour of the whole limb from below the ancle, sustaining the inferior fragment to the top of the leg. The principle, in short, was that of the laced stocking, modified to act especially on a definite point and space. The compressing bandage was inspected, and generally tightened twice a day, and always drawn as firmly as the patient's feelings would allow. The limb was kept extended and in splints, to maintain steadiness, and preserve the correct axis of the foot on the leg.

"The favourable tendency of this treatment soon became manifest. In ten days the chasm or interval of the edges of the bone along the line of fracture, was much diminished, the bones having sensibly approximated by absorption of the interposed deposit. After three weeks the depression which had always marked the line of fracture, and extent of separation, was obliterated to the touch, and the ends of bone seemed in close contact. Now, too, the tendency to eversion of the foot was lost, and moderate lateral pressure on the foot did not cause any parting at the fissure. A further particular detail is unnecessary, perseverance in the course described, produced at the end of five weeks from its adoption, unequivocal bony union. At that period the patient could elevate the whole limb; flex, extend, and rotate the foot freely, without the slightest evidence of motion in the line of fracture. The splints were thrown off, but the compressing bandage directed to be worn less firmly applied for some weeks, and the patient admonished to avoid for the present throwing the weight of the body on the affected limb.

"The cure in this case became perfect, and when the limb was enough recovered to take its share of weight and motion, it was found to have shortened less than one-fourth of an inch. The man walks now without halting, and as firmly and well on one limb as the other. This patient was confined to bed nineteen weeks and two days; and it was six months from the accident, before the consolidation was sufficient to permit use of the limb in walking.

Case 2nd. was that of a man 60 years old. Both the bones of his leg were broken, and the tibia nearly projected through the skin. The fibula united, but no union of the tibia took place under the ordinary treatment. The patient had been intemperate; and, previously to the accident, the leg was in a state of chronic enlargement, from infiltration and thickening of the cellular substance. Under these unpromising circumstances,

"It was determined to try strict and continued pressure. With this intention, a roller was laid regularly from the toes to the knee. Over this was applied short splints, made of binder's boards cut to suit the outline of the limb, then macerated, that they might be made to adapt themselves intimately to the surface about the fracture. Those splints were laid, while pliable from wetness, on opposite sides of the fracture, and braced in their place, as firmly as could be borne, by a second roller, carried from the ancle to the top of the leg. Two latteral splints of the same material, not macerated, were next applied and secured at intervals by a few turns of a roller. These splints were sufficiently long to steady the leg, and prevent motion of the foot, the wood splints first used were laid aside, and the patient confined to bed, with the limb extended. An occasional change from the recumbent to the sitting posture, was allowed the patient; but he was altogether prohibited from getting out of bed or bringing the limb into use.

"An evident change in the relation and aspect of the parts, was observed in a fortnight after this course was instituted. When the plan of compression was adopted, the superior fragment of the tibia projected so sensibly at the point of separation, as to cause much deformity by its remarkable prominence. At the end of two weeks, this projection was much reduced, and by the fourth week, the bone had regained its natural level; doubtless, by the influence of pressure on the deposited matter, which had occupied the interval between the fractured surfaces. The facility and range of motion at the fracture, were also lessened though still discoverable. The pressure, together with confinement to position, were steadily maintained for six weeks. The long splints were then taken off, and the patient permitted to put the limb to the floor, and, supported by crutches, to bring it cautiously into use. The roller and close splints were kept on some time longer.

"When the point of fracture was examined, some weeks after the patient began to use the limb, the part appeared very firm. No pressure, which it was thought proper or necessary to employ, whether directed immediately to the fracture, or by using the foot as a lever to rotate the limb freely, caused the slightest indication of disunion. In a month or six weeks after the limb had been brought into use, the patient threw aside all artificial supports, and walked as well as before the accident. There is no discoverable shortening of the leg.

Case 3. This was a fracture of the ulna. At the end of five weeks, not the least union had taken place.

"Pressure was resorted to in this instance also. A roller was laid smoothly from the fingers to, and some distance above the elbow. Short splints, of binder's board, were fitted to the interior and external surface of the limb, to afford sufficient lateral support, and were well secured in place by a roller. A long splint of binder's board, incurved, and adapted to the arm, was laid along its ulnar line, extending below the wrist to support the hand, and a shorter splint of the same material rested on the radial side, the two forming an external case far the arm, not quite close, or meeting at the edges. The splints were made to embrace the arm by straps at short spaces, which, particularly at and near the fracture, were drawn as tight as could be borne. A due degree of pressure was thus constantly maintained as long as necessary, and the result was satisfactory in this instance as in the preceding cases.

